# Assessment of beliefs and attitudes about electroconvulsive therapy posted on Twitter: An observational study

**DOI:** 10.1192/j.eurpsy.2022.2359

**Published:** 2023-01-09

**Authors:** L. de Anta, M. A. Alvarez-Mon, C. Donat-Vargas, F. J. Lara-Abelanda, V. Pereira-Sanchez, C. Gonzalez Rodriguez, F. Mora, M. A. Ortega, J. Quintero, M. Alvarez-Mon

**Affiliations:** 1 Department of Psychiatry and Mental Health, Hospital Universitario Infanta Leonor, Madrid, Spain; 2 Department of Medicine and Medical Specialities, University of Alcala, Madrid, Spain; 3 Ramón y Cajal Institute of Sanitary Research (IRYCIS), 28034 Madrid, Spain; 4 ISGlobal, Barcelona, Spain; 5 CIBER Epidemiología y Salud Pública (CIBERESP), Madrid, Spain; 6 Unit of Cardiovascular and Nutritional Epidemiology, Institute of Environmental Medicine, Karolinska Institute, Stockholm, Sweden; 7 Departamento Teoria de la Señal y Comunicaciones y Sistemas Telemáticos y Computación, Escuela Tecnica Superior de Ingenieria de Telecomunicación, Universidad Rey Juan Carlos, 28942 Fuenlabrada, Spain; 8 Department of Child and Adolescent Psychiatry, NYU Grossman School of Medicine, New York, New York, USA; 9 Centro de Salud Mental Infanto Juvenil Cornellá, Hospital Sant Joan de Deu, Barcelona, Spain; 10 Department of Legal and Psychiatry, Complutense University, Madrid, Spain

**Keywords:** ECT, electroconvulsive therapy, public opinion, social media, Twitter

## Abstract

**Background:**

Electroconvulsive therapy (ECT) is an effective and safe medical procedure that mainly indicated for depression, but is also indicated for patients with other conditions. However, ECT is among the most stigmatized and controversial treatments in medicine. Our objective was to examine social media contents on Twitter related to ECT to identify and evaluate public views on the matter.

**Methods:**

We collected Twitter posts in English and Spanish mentioning ECT between January 1, 2019 and October 31, 2020. Identified tweets were subject to a mixed method quantitative–qualitative content and sentiment analysis combining manual and semi-supervised natural language processing machine-learning analyses. Such analyses identified the distribution of tweets, their public interest (retweets and likes per tweet), and sentiment for the observed different categories of Twitter users and contents.

**Results:**

“Healthcare providers” users produced more tweets (25%) than “people with lived experience” and their “relatives” (including family members and close friends or acquaintances) (10% combined), and were the main publishers of “medical” content (mostly related to ECT’s main indications). However, more than half of the total tweets had “joke or trivializing” contents, and such had a higher like and retweet ratio. Among those tweets manifesting personal opinions on ECT, around 75% of them had a negative sentiment.

**Conclusions:**

Mixed method analysis of social media contents on Twitter offers a novel perspective to examine public opinion on ECT, and our results show attitudes more negative than those reflected in studies using surveys and other traditional methods.

## Introduction

Electroconvulsive therapy (ECT) is an effective and safe medical procedure with now more than 80 years of use for the treatment of different mental disorders [1, 2]. In fact, about 1.4 million people are treated every year with ECT worldwide, being treatment-resistant depression its most frequent indication [3]. Reported remission rates in literature are 52% in randomized clinical trials and 75% in open trials, which are big figures taking into account that ECT is used as a rescue after several failed medication trials [4, 5].

Despite its demonstrated effectiveness, safety, and rapid action, ECT has been for long one of the most controversial procedures in medicine [6]. Views among people without specialized knowledge and experience can indeed be very negative: a study has shown 63% of people disclosing negative perceptions toward ECT, and in another study, 75% of interviewees considered this procedure unsafe [7–11]. Such public views, reflecting lack of knowledge and stigma, can negatively influence the consent of people with mental disorders who could potentially benefit from it. Those views are fed by contents on the media. In fact, media has traditionally portrayed ECT as an abusive and dehumanized treatment, and of little therapeutic benefit [12–14]. Controversy on ECT extends to healthcare providers, among which misinformation contributes to negative views [15–17].

Up to the date, the main methods to study public opinions and attitudes toward ECT have been “traditional,” mainly including information from clinical trials and surveys and questionnaires administered during the medical consultation [18–21]. Those kinds of studies, particularly focused on people with lived experience and relatives, have been calling for new methods to more accurately approach opinions not only in those populations but also among the general public, healthcare professionals, media, and other social actors. In these regards, research focused on social media contents has been emerging in the last years as a tool to more naturalistically and thoroughly capture opinions [22, 23]. This novel approach is also able to include those individuals reluctant to fill out questionnaires and surveys [24]. Moreover, as social media discussions happen in settings more casual and spontaneous than healthcare appointments and formal surveys, they are more likely to reflect sincere views and beliefs [25–28].

The aims of this study were to (a) identify social media contents on Twitter related to ECT; (b) characterize the types of users and contents involved in those publications; and (c) analyze the interest and sentiment of such publications by users and topics.

## Materials and Methods

### Collection of Twitter data and content analysis process

This mixed method quantitative and qualitative analysis of social media contents focused on tweets related to ECT. Our inclusion criteria were (a) public tweets; (b) containing readable text in English or Spanish; (c) using any of the keywords “electroconvulsive therapy,” “electroshock,” or their Spanish equivalents anywhere in the tweet; and (d) published between January 1, 2019 and October 31, 2020, a wide time frame aiming to capture broad and usual social media discussion on the topic.

The tool we used to collect tweets is Tweet Binder, which we have extensively used in past research and is able to access 100% of public tweets within a given framing limit in terms of keywords, such as time, of a search query. Apart from the tweet texts, this tool provides the retweet and like count for each tweet, as well as their time and date, permanent link, and user description. The query led to a collection of a total of 31,783 tweets.

**Figure 1.**
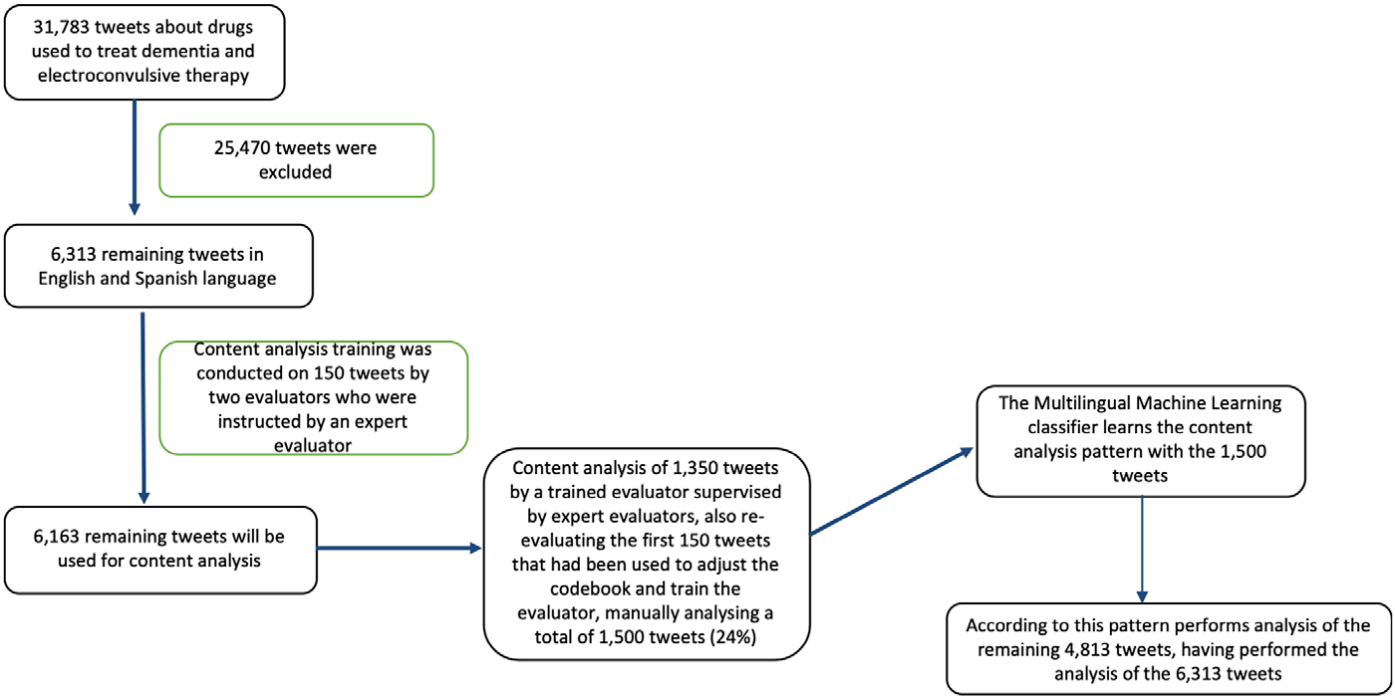
Tweet analysis flowchart.

Content analysis used a semi-supervised machine-learning approach with three phases. First, a filter was applied to remove tweets that did not match all inclusion criteria (*n* = 25,470 were excluded due to them being in different languages or written in a non-self-explanatory way which made their meaning uncertain). Second, with the remaining tweets (6,313), a manual, qualitative classification of a small subset of tweets (*n* = 1,500) by investigators. Third, an automated computerized classification of the remaining, larger subset of tweets (*n* = 4,813) based on the topical categories created in the first phase and on sentiment analysis software. All the process is illustrated in Figure 1.

### Exploration of data and identification of topical categories

We used a mixed inductive–deductive approach to develop a codebook to classify tweet contents based on key topical categories (i.e., codes). Deductively, we brought categories from previous Twitter research from our team [29, 30]. Inductively, we explored an initial subset of 150 tweets (from the small, manual classification subset) to identify potential new topics and refine the codebook. For that, two investigators (LdA and CGR) separately coded those 150 tweets, discussing their discrepancies and reaching a final consensus coding *a posteriori* with the mediation of a third investigator (MAA-M). Once the final codebook was agreed upon, those two investigators coded the remaining 1,350 from the first subset.

The codebook combined a hierarchical and a parallel structure. We first divided tweets between “medical” and “nonmedical.” “Medical” tweets would be subclassified into subcodes depending on the context in which ECT was discussed about “depressive mood or bipolar disorder,” “psychotic symptoms, catatonia, or other areas of interest,” “special situations” (including pregnancy, comorbid conditions, and old age), “cognitive complaints” (in terms of memory loss, confusion, functional impairment, etc.) or “medical-unspecified.” “Nonmedical” tweets were subclassified in “commercial activities” (including financial, marketing, promotion, or legal issues), “education or divulgation” (related to conferences, educational activities, books, etc.), “request, offer, or thanksgiving,” and “trivializing” (including jokes, stigmatization, vulgarity, etc.). Additionally, regardless of their “medical” or “nonmedical” content, personal opinions were coded as positive or negative.

Finally, Twitter users for the included tweets were classified into five user types: “people with lived experience” (those users who describe their experience with ECT), their “relatives” (including family members and close friends or acquaintances), “healthcare professionals” (including healthcare institutions), “media” (press, radio, TV, etc.), and “other” (unspecified as not fitting the previous types). Classification criteria and examples of tweets are shown in Supplementary Material.

### Machine-learning classifier

The second, larger subset of tweets (*n* = 4,813) was classified into the codebook categories through machine-learning software, to allow for automated analysis of large amounts of tweets. We used semi-supervised learning. In our case, we use a previously trained transformer and fine-tuned it with the 1,500 manually classified tweets. The first step was preprocessing. We used the *nltk.emoji* and *TweetNormalizer* libraries to normalize the tweets. The *nltk.emoji* [31] library transforms emojis into text (e.g., it would transform a “smiley face” into the text “smiley face.” The *TweetNormalizer* library [32] processes the tweet and transform web links into the text “URL,” the mentions to other Twitter accounts to “@USER,” and delete long blank spaces. As we used a language transformer that have been trained in English, we had to translate all the Spanish tweets, which we did through the *googletrans* library. Finally, we divided our dataset into train and test sets for fine-tuning 70/30 proportion. Additionally, we used the *BERTweet* transformer, which is the first public large-scale language model pretrained for English Tweets. *BERTweet* is trained based on the *RoBERTa* pre-training procedure. The corpus used to pretrain *BERTweet* consists of 850 million English tweets [33]. In order to choose the hyperparameters, we used a validation set of 20% of the train set to select the number of learning rate and batch size.

### Statistical analysis

We estimated the tweet frequencies by several characteristics of the tweets. Comparisons of number of tweets between different characteristics were carried out using Pearson’s chi-square test. We also compared number of retweets and likes per tweet between categories using the ANOVA. A *p*-value less than 0.05 was considered statistically significant. Analyses were conducted with the software packages STATA v16 (StataCorp) and MS Excel.

## Results

### Healthcare professionals are the most active specific type of users

From the 6,313 tweets that were coded, a total of 5,369 tweets fell into the codebook categories, and the rest (*n* = 944) were considered unclassified and thus excluded. As for types of users, most were “other” (unspecified, 63%) while the predominant types among the specified users were “healthcare professionals” (25%) followed at distance by “people with lived experience” (9%), “media” (2%), and “relatives” (1%).

Retweet and like counts per tweet per type of user are depicted in Table 1. Those measures of elicited public interest were highest in terms of retweets for “media” (17/tweet) and “healthcare professionals” (16/tweet), and highest by far in terms of likes for “people with lived experience” (114/tweet), and “relatives” (72/tweet).

**Table 1.** Retweet and like counts per tweet by different categories classified: Types of users, medical tweets, nonmedical tweets, and personal opinion.

	Retweets	Likes
Types of user		
People with lived experience	8.6	114.2
Relatives	6.6	72.6
Healthcare professionals	16.0	48.2
Media	17.1	27.0
Other	8.3	46.3
Medical tweets		
Depressive mood or bipolar disorder	13.9	47.2
Psychotic symptoms, catatonia, or other areas of interest	13.1	43.0
Special situations	11.0	44.9
Cognitive complaints	14.1	77.3
Nonmedical tweets		
Other	12.6	50.8
Commercial activities	11.9	11.2
Education and divulgation	15.9	45.5
Request, offer, or thanksgiving	29.9	39.7
Trivializing	7.7	59.5
Personal opinion		
Negative	18.93	66.012
Positive	12.139	119.784

### Medical content is scarce, and in most cases, it is posted by healthcare providers

**Figure 2.**
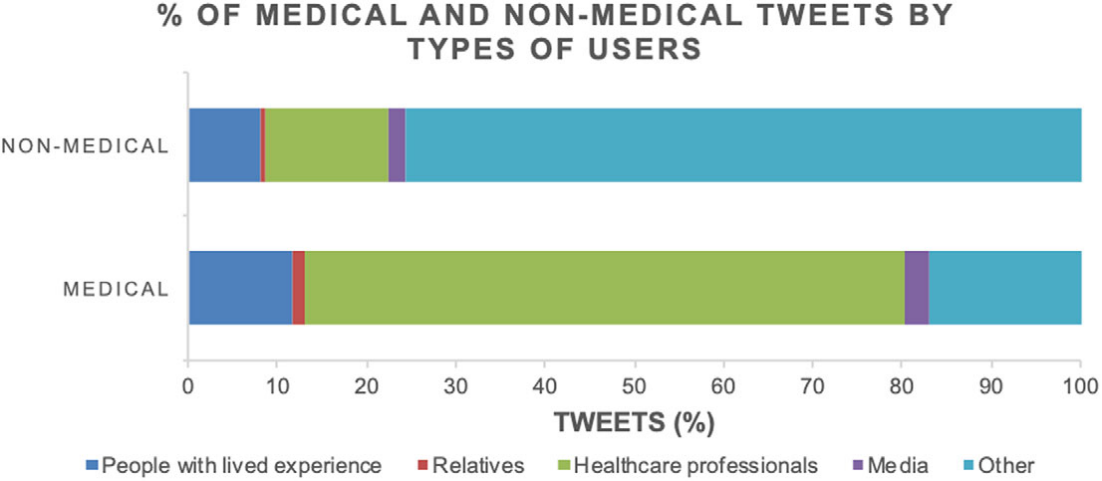
Percentage of “medical” and “nonmedical” tweets by types of users.

**Figure 3.**
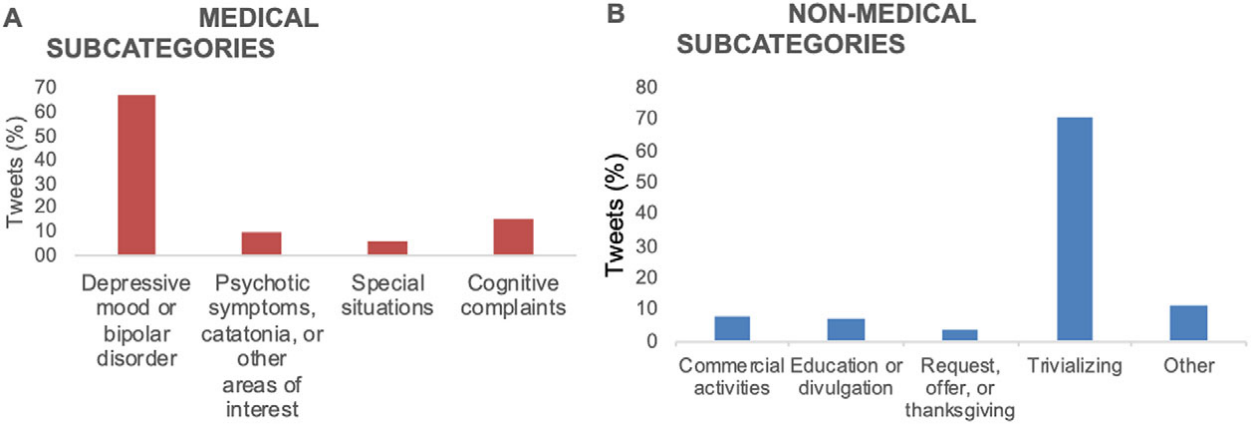
Percentage of tweets by “medical” subcategories (A) and “nonmedical” subcategories (B).

“Medical” tweets were less abundant (1,134/5,369, 21.12%) than “nonmedical” (4,235/5,369, 78.88%). Moreover, 67.11% of those were published by “healthcare professionals” and 11.64% by “people with lived experience,” with a very small percentage (2.91%) by the “media,” as shown in Figure 2. As for “medical” subcategories, “depressive mood or bipolar disorder” was the most frequent (66.4%), followed by “cognitive complaints” (15.6%), “psychotic symptoms, catatonia, or other areas of interest” (9.4%), and “special situations” (5.9%) (Figure 3A).

Retweet and like counts per tweet per “medical” subcategory are depicted in Table 1. “Cognitive complaints” had the highest counts for both: 14.1/tweet for retweets (very slightly higher than for other subcategories) and 77.35/tweet for likes (markedly higher than for other categories by a differential count of 30 points).

### Nonmedical content tweets predominated, the majority being trivializing

As for “nonmedical” tweets, “other” users published most of those, followed by the specified categories “healthcare professionals” (13.8%), “people with lived experience” (8.1%), “media” (2.0%), and “relatives” (0.5%). Figure 3B shows the relative distribution of tweets among “nonmedical” subcategories. “Trivializing” was by far the most frequent type of nonmedical content (70.53%).

**Figure 4.**
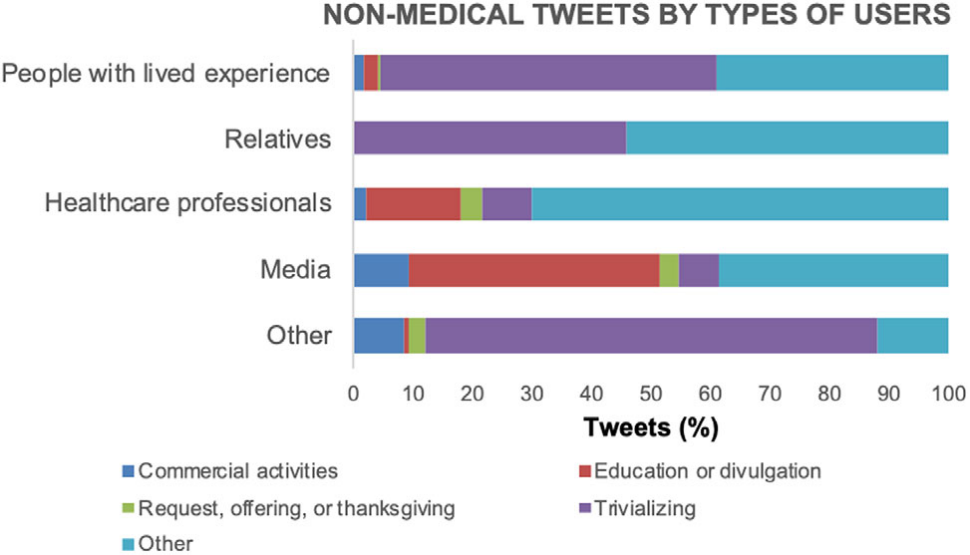
Percentage of “nonmedical” tweets by types of users.

Regarding the types of users authoring these “nonmedical” tweets (Figure 4), we want to note that “others” had the highest relative proportion of “trivializing” contents among the tweets they posted, followed by high percentages of those tweets in “people with lived experience” (56.42% of their “nonmedical tweets”) and “relatives” (45.71%), and lower percentages in “healthcare professionals” (8.54%) and “media” (6.72%).

Retweet and like counts per tweet for “nonmedical” are shown in Table 1. Retweet counts were highest among “request, offer, or thanksgiving” (29.93/tweet), while like counts were highest among “trivialization” (59.46/tweet).

### People with lived experience’ showed greater proportion of positive opinions about ECT than other users

**Figure 5.**
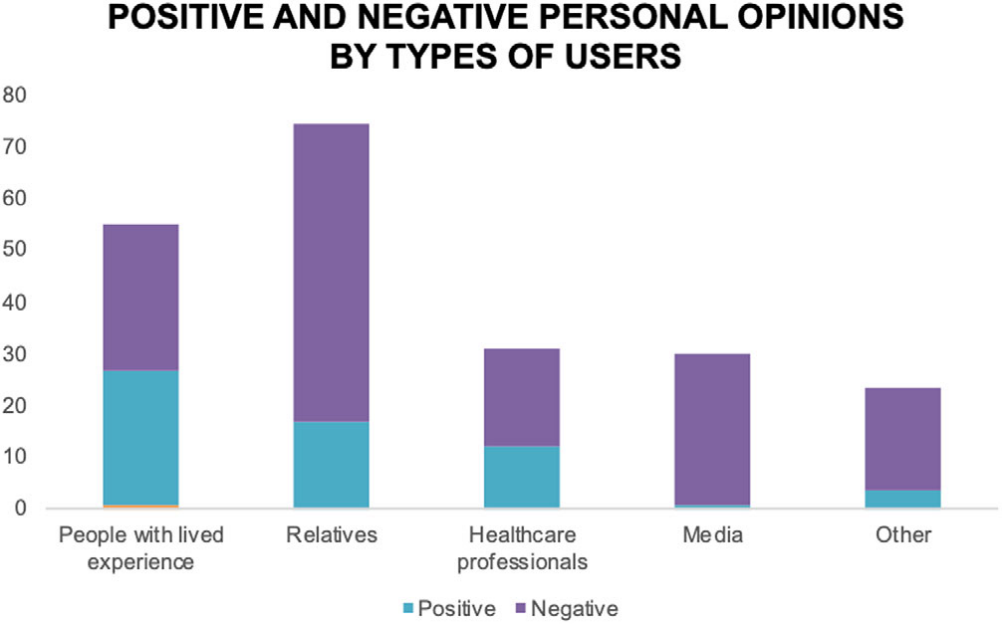
Percentage of “personal opinion” tweets with positive and negative sentiment per type of user. Percentages are calculated over the total tweets for each type of user; blank spaces filling up to 100% in each column correspond to the percentage of tweets not containing a “personal opinion.”

Personal opinions were present in 28.65% of analyzed tweets; most of those had a negative sentiment (20.9% of analyzed tweets, versus 7.75% with positive sentiment) in regards to ECT. Stratifying per type of users, as shown in Figure 5, “people with lived experience” had very similar percentages of positive and negative opinions (25.68% positive and 28.21% negative, both percentages over the total tweets from these users), while “relatives” had about three more times negative than positive opinions, “healthcare professionals” had a slightly higher proportion of negative (18.8%, versus 12.04% positive, both percentages over the total tweets from these users), and “media” users had the highest differential between negative and positive opinions, with virtually all of their opinions being negative (29.41%, versus 0.84% positive, both percentages over the total tweets from these users).

As for retweet and like counts per tweet depending on their opinion, we found that negative opinions had higher retweet per tweet counts (18.93/tweet) than positive opinions, but lower like to tweet counts (Table 1).

## Discussion

Our results show that people with lived experience and their relatives do not seem to be very active in Twitter discussions regarding ECT, while healthcare professionals and institutions are the most active users, and their contents focus on medical aspects. In terms of medical areas discussed on Twitter, tweets regarding depressive and bipolar disorders are more frequent than those related to cognitive complaints, yet the latter elicited more interest. Overall, nonmedical topics were more frequent than medical ones, and more than half the tweets trivialized ECT. We also observed that personal opinions regarding ECT were mostly negative among all types of users, which should be particularly concerning in the case of patients and all the more of healthcare professionals.

When discussing this study in the context of previous research on mental health topics on Twitter, it is noted that the total amount of retrieved publications for ECT is lower than the amount of retrieved tweets in other studies related to other treatments for mental disorders including antipsychotics, antidepressants, and medications for attention-deficit/hyperactivity disorder [27, 28, 34]. They are even lower in amount than tweets for nonpsychiatric disorders, such as statins, medications for obesity, chemotherapy agents, or antibiotics [24, 34–36]. Such differences seem coherent with the lower prevalence of ECT use as compared to those other treatments.

The aforementioned previous Twitter research related to psychopharmacological drugs also described the types of users involved in discussions related to such treatments [28, 34]. For antidepressants and antipsychotics, people with lived experience and their relatives were authors of more than half the tweets, which contrasts with the lower relative proportions of tweets from such users in ECT tweets in our study. This is surprising to us, as we expected more similar, higher percentage of tweets by these types of users, as ECT is a treatment which primary target disorders are the same as for those medications (depression and schizophrenia), yet used as a second or third line of treatment in case of resistance to such psychopharmacological interventions. Our expectation was also based on previous research showing that Twitter promotes discussions related to mental health, that people with depression seem eager to share about their condition on online platforms, and that people with psychotic-spectrum disorders frequently resource to online platforms to meet people with same conditions [37–40]. We expected that people with lived experience would be as likely eager to talk about ECT than to do so about medications. We propose two explanations for such findings. First, our understanding of ECT as a highly stigmatized treatment, which would prevent people from discussing own or close personal experiences related to it, despite the potential anonymity that Twitter offers. Another speculative explanation is that in the case of medications, there are more options to choose—for instance, in terms of differential mechanism of action or tolerability among drugs for depression or psychosis—what might make people eager to find experiences and opinions from other people with lived experience on social media; by contrast, ECT is usually prescribed in situations where there are no other options to be considered and compared.

The finding that depression and bipolar disorders are the most discussed medical issues in these tweets is consistent with the actual main indications of ECT [41, 42]. It is somewhat unexpected, the relative paucity of tweets discussing cognitive complaints with ECT, because those complaints are the most commonly reported and studied side effect of this treatment [43, 44]. As for “nonmedical” contents, those we deemed as “trivializing” were the most abundant, what is remarkable and seems disproportionate based on the established effectiveness and safety of ECT [41, 42]. This abundance is relevant, as joking and stigmatizing contents influence opinions and attitudes among the general public, people with mental disorders, and healthcare professionals [45, 46]. Previous Twitter research has shown frequent trivialization also present in nonpsychiatric health conditions such as epilepsy and AIDS, but mental disorders seem to be more subject to stigmatizing attitudes [47–49]. A study published in 2019 showed that pejorative tweets were much more frequent in psychosis (36.3%) than in breast cancer (15%), diabetes (12.75%), Alzheimer’s (7.6%), or HIV (2.72%) [50]. Another study in the same year compared trivialization between mental disorders and physical health conditions, demonstrating that such contents were 2.10 higher in the former [47]. Our research team has analyzed in the past stigmatizing attitudes regarding psychopharmacological treatments finding notable proportions of such contents, which resemble proportions found for discussions on mental disorders [28, 34]. This leads us to think that stigmatization of mental disorders extends to psychopharmacological treatments [50], and it is remarkable that such stigma seems much higher for ECT, being present in more than half of their tweets of our current sample.

Moreover, it is noteworthy that such joking and stigmatizing contents regarding ECT on Twitter are not only published by unspecified users (which seem to reflect the negative social opinion as reflected in previous research), but also by people with lived experience and their relatives, and, all the more concerning, by healthcare professionals and institutions. We relate this to other past research findings showing negative views on ECT in the healthcare sector including medical students and even psychiatrists [51, 52]. This seems to be owed to lack of education on this treatment, which is susceptible to improvement with training [53, 54].

Our research brings also a novelty in the field of exploration of social attitudes reflecting personal opinions regarding ECT. Abundant previous research has aimed at improving knowledge on public views toward ECT and its controversies, particularly focusing on discussions by people with lived experience, and mostly showing positive views among them [55–57]. In a study carried out in Dublin, published in 2007, 88% of participants said that they would be willing to receive ECT, and 71% reported improvement with this treatment [58]. Another study, conducted earlier in Turkey with patients with bipolar disorder who had received ECT, showed similar results, with 78.6% of participants reporting benefits from the treatment [59].

Our study, however, shows that people with lived experience published a slightly higher amount of negative than positive opinions. Our study is the first exploring such opinions on ECT through a mixed-method analysis of social media publications, and we believe that this method can approach better the complexity of public opinions. Furthermore, Twitter allows for spontaneous conversations and potential anonymity, reducing the risk of social desirability biases [60]. Indeed, a study in England, published in 2003, concluded that ECT users were more prone to provide positive opinions when asked shortly after receiving the treatment, in hospital settings, and by the treating physician [61]. Our findings of higher proportions of negative opinions on social media versus those reported by previous research using traditional methods have also been observed in explorations of opinions for certain medications: for example, research on methotrexate for rheumatoid arthritis found that doctors, especially rheumatologists, received mostly positive comments from patients about their treatment, while publications on social media and online are predominantly focused on bad experiences [62]. In the case of relatives of ECT users, previous studies have reported mostly positive attitudes and satisfaction, which contrast with our findings, probably due to similar reasons as it happens with people with lived experience [21, 59].

Finally, it is worth noting the proportion of negative opinions among healthcare professionals and institutions. As it happens with stigmatizing contents, previous research relates this to misinformation among professionals unfamiliar with ECT, which improves with education. Regardless of their scientific education and professional training, these persons are subject to the influence of media and misinformation as any other individual, and might have formed their own opinion about ECT previous to their healthcare education. This renews calls for efforts to increase and improve knowledge about ECT among these professionals, so they can disseminate accurate information and combat stigma.

### Limitations

This study has some limitations. First, Twitter users tend to be younger than the general population, so predominant contents on this social media platform might not coincide with the main areas of interest in discussions on ECT off Twitter. Second, the intrinsic limitation of our units of content analysis (tweets, which are by definition very short) made it impossible to obtain information from many potential areas of interest, including other medical topics. Third, while our tweet search and collection tool is able to identify all tweets for a specific query, it is possible that our inclusion criteria left out relevant tweets related to ECT but not using our keywords. Notwithstanding all these limitations, we used a methodology that has been consistently employed in past Twitter research.

## Conclusions

This mixed-method analysis of Twitter posts provides a novel and insightful approach to public opinions regarding ECT; an approach that addresses social desirability biases from traditional survey methods by observing spontaneous discussions on a platform not conditioned by clinical supervision and closed-ended questions. We found that personal opinions regarding ECT on Twitter seem more negative than what has been observed by traditional survey methods, reflecting fears and prejudices among people with mental disorders and their relatives in a way that is very relevant for psychiatrists to consider when prescribing this treatment. Such high levels of negative opinions and stigmatizing contents among all types of Twitter users suggest that lack of knowledge and misinformation about ECT are common, and further education about it, especially to healthcare professionals, is sorely needed. We consider of paramount importance that knowledgeable psychiatrists participate in social media conversations related to this disorder so they can address questions from the public and dispel myths, addressing misinformation and stigmatizing publications, and, making sure they do not contribute to those, which would create further confusion.

## Data Availability

The raw data supporting the conclusions of this article will be made available by the authors, without undue reservation.

## Supplementary material

For supplementary material accompanying this paper visit http://doi.org/10.1192/j.eurpsy.2022.2359. click here to view supplementary material

## Supplementary Materials

To view supplementary material for this article, please visit http://doi.org/10.1192/j.eurpsy.2022.2359.
