# Supplementary material for: Assessment of beliefs and attitudes about electroconvulsive therapy posted on Twitter: An observational study
Source: Eur Psychiatry. 2023 Jan 9;66(1):e11. doi: 10.1192/j.eurpsy.2022.2359 (PMC9970148; doi:10.1192/j.eurpsy.2022.2359)
Supplement: Supplementary file 1 [file S0924933822023598sup001.pdf]

Supplementary Material. Table: Category, definitions, and examples of tweets. Usernames and personal names were removed.

Category	Definition of category	Example tweet
**Medical content**		
**Depressive mood or bipolar disorder**	The tweet refers to depressive mood or symptoms related to bipolar disorder such as manic or hypomanic states as an area of interest related to ECT	*How Electroconvulsive Therapy (ECT) Helped Me Survive to Battle Bipolar Depression*
**Psychotic symptoms, catatonia, or other areas of interest**	The tweet refers to psychosis or other psychosis-related symptoms as well as catatonia as an area of interest related to ECT	*@XXX Dude, are you totally insane or just a prisoner of your circumstances? Whatever it is, you need to urgently see a Psychiatrist. Electroconvulsive Therapy (ECT) can sometimes do wonders for curing Psychosis. Plz give it a go. It might reset your brain. I'll even pay for it. Go On*
**Special situations**	The tweet refers as an area of interest related to ECT in special situations such as pregnancy, comorbid conditions, or old age.	*Current electroconvulsive therapy practice and research in the geriatric population. This is very interesting! https://t.co/1AZ1i68oci*
**Cognitive complaints**	The tweet refers to confusion, memory loss or any other cognitive impairment as an area of concern related to ECT	*i keep seeing "things i noticed at my lw" posts and bitch at my lowest weight i was so unwell that i needed 30 induced seizures aka electroconvulsive therapy to save my life which also robbed me of 2 years of memories, mental illness isn't a fucking trend or a fashion statement !*
**Non-medical content**		
**Commercial activities**	Tweets discussing financial, marketing, promotional, or legal issues	*#PHYSICAL #THERAPIST JOBS Denver CO USA - RN/Registered Nurse ECT Electroconvulsive Therapy - Assumes responsibility and accou: Assumes responsibility and accountability for facilitating communicating and collaborating with both the healthcare team https://t.co/VB6I5urwTV*
**Education or divulgation**	Tweets related to conferences, educational activities, books, etc	*Current Issue: Feasibility of the Audio Recorded Cognitive Screen in the Assessment of Individuals Undergoing Electroconvulsive Therapy https://t.co/chTIN2Y5RC*

		Handbook of ECT: A Guide to Electroconvulsive Therapy for Practitioners (English Edition) https://t.co/7ewWzhOJ6N #洋書 #Medical Books
**Trivialization**	Tweets that include jokes, stigmatization, vulgarity, etc.	*@XXX He gets an electroshock his brain if he says anything negative about Putin?*<br><br>*Idea for the #Debates2020 put both people in separate booths onstage. When one person has the floor cut the other booths mic so no one can hear them. Or administer a mild electroshock to whomever speaks out of turn. Obviously less safer but arguable higher entertainment value.*
**Positive personal opinion**	Tweets that include a positive personal opinion about ECT	*Electroshock therapy has been going great* 🤤
**Negative personal opinion**	Tweets that include a negative personal opinion about ECT	*do you know that mental hospitals still perform electroshock to the brain as a form of "therapy"? ECT is torture not treatment! #banECT*
**Type of user**		
**People with lived experience**	Tweets posted by users who describe their experience with ECT	*How Electroconvulsive Therapy (ECT) Helped Me Survive to Battle Bipolar Depression*
**Relatives**	Tweets posted by family members and close friends or acquaintances.	*@XXX @XXX @XXX @XXX @XXX @XXX A few weeks ago I shared my family's story of #schizophrenia. Despite that every #patient is different, I saw first hand the before and after results of #electroconvulsive therapy; #ECT on my sister = acute #catatonia and #loss of #speech for 2 years: https://t.co/xfppwPw1Xm*
**Healthcare professionals and healthcare institutions**	Tweets posted by healthcare professionals (including students or professionals in	*Interesting thing I learned this yr during med school was that electroconvulsive therapy is very safe (even in pregnant women & elderly) & effective in treatment-resistant depression. Done w/ muscle relaxant & anesthesia. @XXX special The Great Depresh destigmatizes it*

	training) or health institutions	
**Media**	Tweets posted by press, radio, television or news platforms.	*Good news for those who suffer from depression! The two most widely used forms of brain stimulation therapy—electroconvulsive therapy and transcranial magnetic stimulation—will soon be available at Lakeland Regional Health.*
